# Diatom flagellar genes and their expression during sexual reproduction in *Leptocylindrus danicus*

**DOI:** 10.1186/s12864-017-4210-8

**Published:** 2017-10-23

**Authors:** Deepak Nanjappa, Remo Sanges, Maria I. Ferrante, Adriana Zingone

**Affiliations:** 1 Stazione Zoologica Anton Dohrn, Villa Comunale, 80121 Naples, Italy; 2 College of Earth, Ocean and Environment, University of Delaware, 700 Pilottown Road, Lewes, 19958 Delaware USA

**Keywords:** *Leptocylindrus*, Next generation sequencing, Transcriptome, Diatom, Sexual reproduction, Flagellar genes

## Abstract

**Background:**

Flagella have been lost in the vegetative phase of the diatom life cycle, but they are still present in male gametes of centric species, thereby representing a hallmark of sexual reproduction. This process, besides maintaining and creating new genetic diversity, in diatoms is also fundamental to restore the maximum cell size following its reduction during vegetative division. Nevertheless, sexual reproduction has been demonstrated in a limited number of diatom species, while our understanding of its different phases and of their genetic control is scarce.

**Results:**

In the transcriptome of *Leptocylindrus danicus*, a centric diatom widespread in the world’s seas, we identified 22 transcripts related to the flagella development and confirmed synchronous overexpression of 6 flagellum-related genes during the male gamete formation process. These transcripts were mostly absent in the closely related species *L. aporus*, which does not have sexual reproduction. Among the 22 transcripts, *L. danicus* showed proteins that belong to the Intra Flagellar Transport (IFT) subcomplex B as well as IFT-A proteins, the latter previously thought to be absent in diatoms. The presence of flagellum-related proteins was also traced in the transcriptomes of several other centric species. Finally, phylogenetic reconstruction of the IFT172 and IFT88 proteins showed that their sequences are conserved across protist species and have evolved similarly to other phylogenetic marker genes.

**Conclusion:**

Our analysis describes for the first time the diatom flagellar gene set, which appears to be more complete and functional than previously reported based on the genome sequence of the model centric diatom, *Thalassiosira pseudonana.* This first recognition of the whole set of diatom flagellar genes and of their activation pattern paves the way to a wider recognition of the relevance of sexual reproduction in individual species and in the natural environment.

**Electronic supplementary material:**

The online version of this article (10.1186/s12864-017-4210-8) contains supplementary material, which is available to authorized users.

## Background

Diatoms are among the most important components of the marine pelagial, where they contribute ca 40% of the total production [1]. Diatom blooms fuel the whole trophic web and are responsible for a considerable part of the CO_2_ drawdown in the world’s oceans. In addition to the ecological role of diatom blooms in the oceanic biomass build-up, the enhanced asexual and sexual reproduction rates during population outburst must have profound consequences for the genetic variability and fitness of the individual species, thus influencing evolutionary processes that are at the basis of the formidable success of diatoms [2, 3]. Sexual processes in diatoms are also crucial to the restoration of the cell size. Considerable miniaturization occurs during asexual division due to the peculiar structure of the rigid silica shell, composed of two unequal parts each inherited by daughter cells, which reconstruct new, smaller silica halves within the maternal ones. Therefore, following the high number of mitotic divisions during a bloom (1–2 per day or more), the average cell size considerably decreases, until gametes are formed and conjugation occurs. The soft cell wall of the zygote allows cell enlargement, and a new silica shell is formed only when the maximum size is reached again.

The occurrence of sexual reproduction in diatoms is presumably widespread, and blooms are likely to maximise encounter probability which is necessary to gamete fusion and zygote formation. However, gametes or zygotes or other signals of sexual reproduction have rarely been observed in the natural environment [4, 5], while information on the sexual cycle of diatoms is available for a limited number of species (200 over more than 10,000 known species) mostly through laboratory experiments (see [6] for a review). Even less known are the molecular processes occurring during sexual reproduction. The scarce information available concerns early reports of SIG (sex induced gene) in the centric species *Thalassiosira weissflogii* [7], followed by more refined analyses in the pennate diatoms *Pseudostaurosira trainorii* [8], *Seminavis robusta* [9, 10] and *Pseudo-nitzschia multistriata* [11]. Nowadays the use of genomic tools, boosted by the development of Next Generation Sequencing techniques, offers the possibility to search for genes activated during sexual reproduction and start reconstructing the series of events accompanying the different phases of this crucial process in diatom life cycles.

Interestingly, while in the prevalently benthic pennate diatoms the reproduction is heterothallic and isogamous, in the prevalently planktonic centric diatoms the reproduction is homothallic and large eggs and flagellated male gametes are produced. This is the only case where flagellated stages are observed in diatoms, providing a clear signal of sexual reproduction at least in centric species [6]. By contrast, flagella and cilia are widespread in the vegetative phase of several groups of protists, where they confer motility to the cells and to some extent allow for movements across the water column. Among genes that have been associated to cell motility, those of the intraflagellar transport (IFT) particles are specifically related to the flagellum/cilium development and, along with kinesin and dynein motors, help transport flagellum components from the cell body into the cilium [12]. The IFT particles consist of three subcomplexes called IFT-A, IFT-B and BBSome (Bardet–Biedl syndrome). These subcomplexes are made of many unique proteins and together they are involved in the flagellar assembly, maintenance, and signalling [13–15]. IFT and BBSome genes are either retained or lost in the genomes of the eukaryotic lineages [16]. In diatoms, based on genome sequencing data for the species *T. pseudonana* and *P. tricornutum,* it was put forward that the pennate diatoms have completely lost the flagellum or cilium structure complexes, while the centrics have lost the IFT-A and BBSome subcomplexes, retaining only the IFT-B subcomplex [16].

The worldwide distributed marine centric diatom *Leptocylindrus danicus* [17–19] presents a peculiar sexual reproduction modality, whereby the zygote formed by conjugation turns into a thick walled spore that can resist in a dormancy phase for long time in the sediments. In almost all diatoms the zygote gives rise to vegetative cells instead, while resting stages are formed during asexual reproduction [20, 21]. By contrast, sexual reproduction has not been found in the congeneric *L. aporus,* which despite a high morphological similarity, differs from *L. danicus* in several physiological and ecological characteristics [17, 18, 22–24]. To address functional differences between the two species, we cultivated them and sequenced their transcriptomes taking advantage of the Marine Microbial Eukaryotic Transcriptome Sequencing Project (MMETSP, [25]). The comparison led to the discovery of several genes linked to flagellar development in *L. danicus*. The aim of this study was to test their actual function through qPCR conducted along a sexual event induced in a laboratory experiment. In addition, we exploited the whole transcriptomic dataset produced within the MMETSP [25], where 305 species from 210 genera were sequenced, to search for genes associated to flagellar development in the available diatom transcriptomes and trace phylogenetic relationships across the protist tree based on two flagellar genes.

## Results

### Identification of differentially represented GO terms

The transcriptomes of *L. danicus* (strain SZN-B650) and *L. aporus* (strain SZN-B651) were obtained by sequencing samples collected at the exponential phase, under identical growth conditions for the two species. The transcriptomes of the two species were assembled, annotated and mapped as described in the supplementary material (Additional file 1: Sequence assembly and annotation, and Table S1–Table S4; Additional file 2: Figure S1; Additional file 3: Figure S2; Additional file 4: Figure S3; Additional file 5: Figure S4; Additional file 6: Figure S5). Comparative analysis, following Fisher’s exact test (FDR <0.05), led the identification of 10 GO terms that were identified as differentially represented between the two species (Additional file 7: Table S5A). These classes contained 3917 and 2142 transcripts from *L. danicus* and *L. aporus*, respectively. Nine of the ten GO terms were over represented in *L. danicus*, while in *L aporus* only ‘protein tyrosine kinase activity’ was over represented. We investigated further to obtain lower level GO terms that were differentially represented in the two species transcriptomes (Additional file 7: Table S5B). Among other differences, *Leptocylindrus danicus* showed a higher representation of genes associated with sexual reproduction such as the GO terms ‘cilium’, ‘cilium morphogenesis’, ‘female meiosis’, ‘female gonad development’, ‘female sex differentiation’, ‘development of primary female sexual characteristics’ and ‘sex differentiation’ (Additional file 7: Table S5B), although no specific conditions that could stimulate sexualization had been applied to the culture used for RNA sequencing. Interestingly, in *L*. *aporus* often the genes associated with these GO terms were absent, whereas genes associated with the GO terms ‘androecium development’, ‘stamen development’, ‘stamen formation’ and ‘stamen morphogenesis’ were found, which were almost absent in *L. danicus*. Genes involved in signaling were also over represented in *L. danicus*. These included the GO terms ‘notch signaling pathway’ and ‘smoothened signaling pathway’. The observed differences in the transcriptomes of the two species, in terms of genes associated with the flagellar development necessarily linked to the male gamete formation, paved the way for a deeper investigation into the nature of the annotated transcripts and their expression during sexual reproduction.

### Sexual reproduction and flagellar genes expression in *L. danicus*

**Fig. 1:**
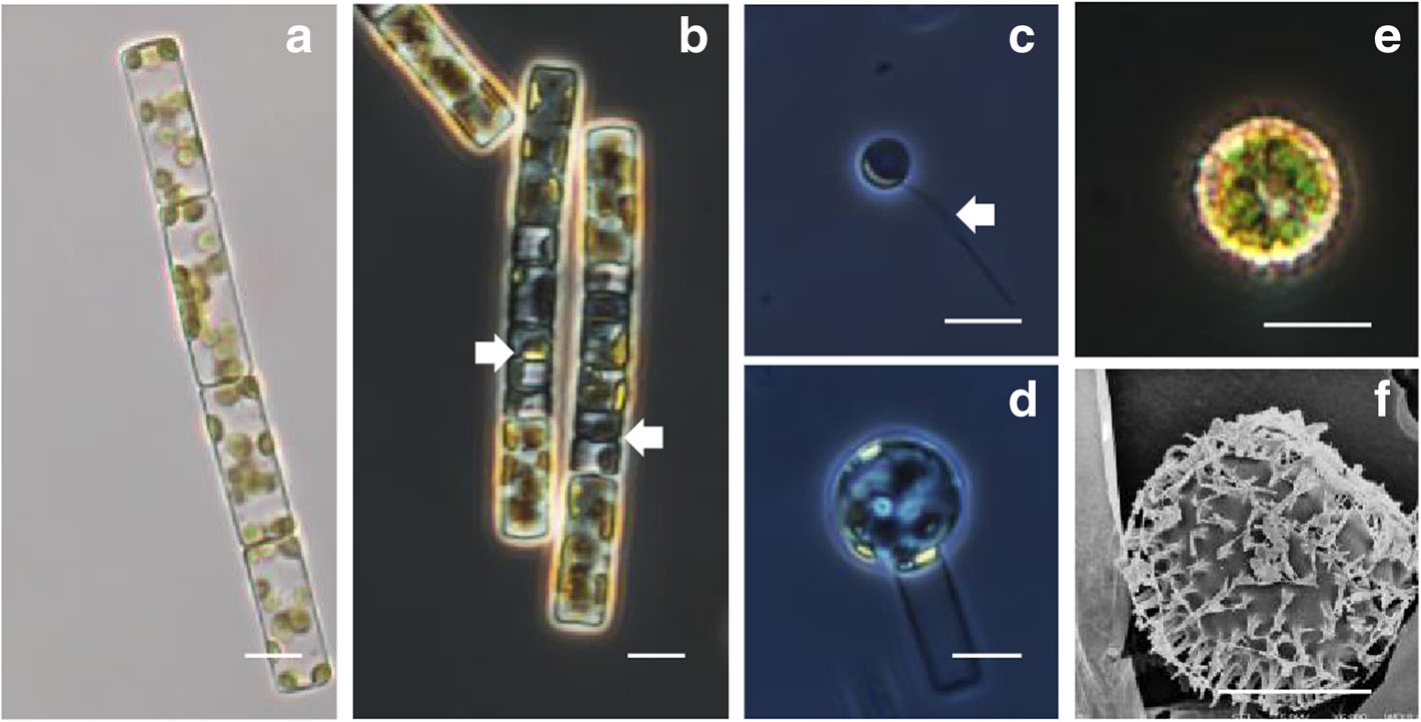
Sexual reproduction stages of *Leptocylindrus danicus*. Scale bars = 10 μm. **a** Epifluorescence micrograph of a chain of four vegetative cells. **b** Cell chains undergoing meiotic divisions, with spermatogonia visible as ‘tetrads’ (arrows). **c** Sperm with one flagellum (arrow). **d** Auxospore in the early developmental stage. **e** Light micrograph of a resting spore. **f** Scanning electron micrograph of a resting spore

Sexual reproduction was induced in *L. danicus* by using a nutrient-depleted, modified F/2 growth medium (T medium) and incubating cells at a slightly lower temperature, 16 °C [26]. In a typical, successful experiment, 36 h from the first subculture in T medium cells started to undergo meiosis, which was recognized by the presence of ‘tetrad cells’ as opposed to cylindrical vegetative cells (Fig. 1a and b). By 48 h most of the cells in the culture were undergoing meiotic divisions, producing gametes within gametangia (Fig. 1c). Gametes were released and auxospores became visible by 60 h (24 h post meiosis initiation), while fully developed resting spores that originate from auxospores were observed after 72 h (36 h post meiosis initiation) (Fig. 1d–f). However, the time taken for the different processes and the proportion of cells undergoing sexual reproduction were variable among induction experiments conducted at different times.

In addition to what occurred upon nutrient depletion and exposure at lower temperature, sexual stages were also observed in cultures grown under normal conditions. With respect to cell size, we did not observe signals of sexual reproduction in cultures with cells having the maximum diameter (>10 μm). Additionally, in maintenance cultures, we observed that cells would automatically undergo cell size expansion after reaching minimal size (<4 μm in diameter) but whether it occurred through sexual processes or vegetative expansion was not ascertained.

**Fig. 2:**
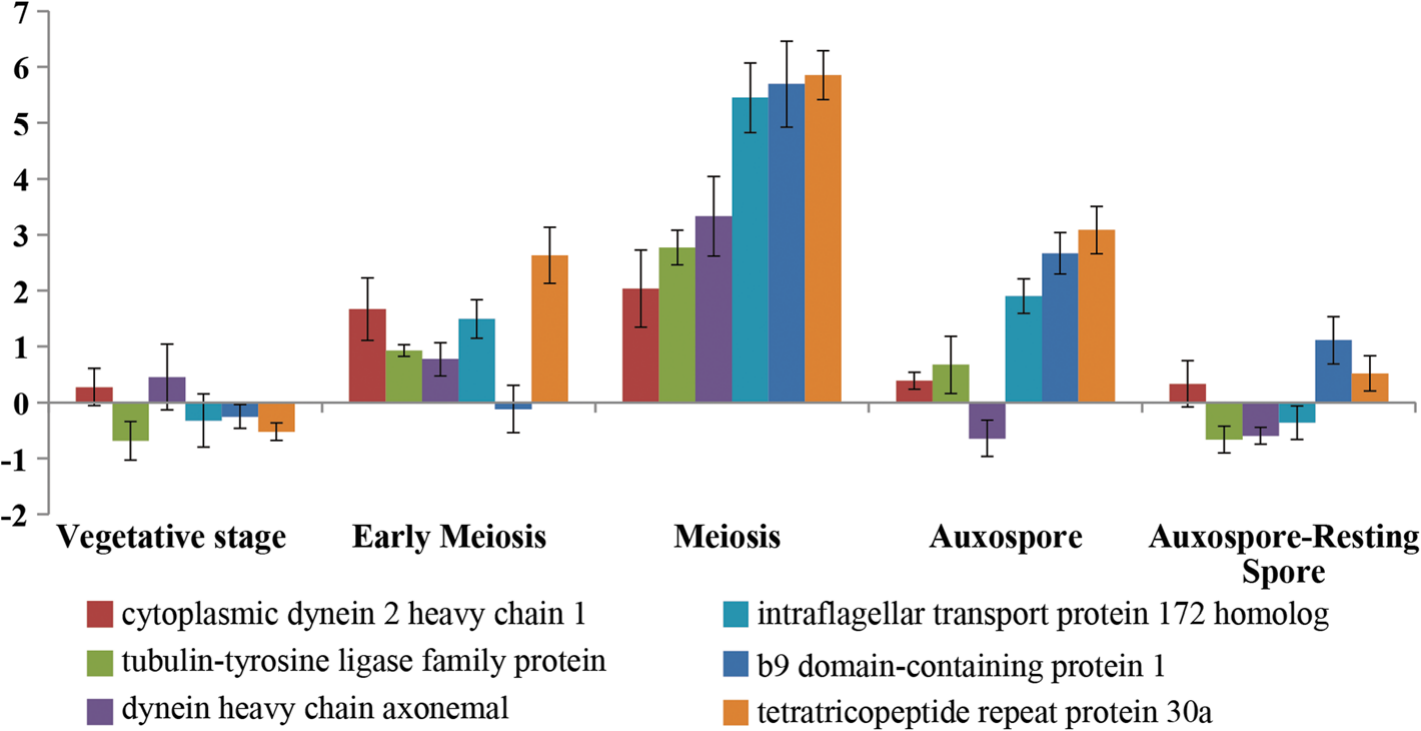
Expression levels of six transcripts related to flagellum at different stages of the sexual reproduction process in *L. danicus*. Values assessed by quantitative RT-PCR and expressed as the fold-increase relative to normalized histone H4 (*n* = 3)

To test specific gene expression during sexual reproduction, we selected six genes that have a core function in the development or the functioning of the flagellum among the 22 transcripts that were associated with the GO terms ‘flagellum’, ‘cilium’, ‘cilium assembly’, ‘motile cilium’ and/or ‘cilium morphogenesis’. The genes selected included: ‘cytoplasmic dynein 2 heavy chain’, ‘dynein heavy chain axonemal’, ‘intraflagellar transport protein 172’, ‘tubulin tyrosine ligase-like 1 protein’, ‘tetratricopeptide repeat protein 30a’, and ‘B9 domain containing protein 1’. The expression levels of these transcripts were assessed through quantitative real-time PCR analysis at four stages of sexual reproduction: early meiosis (12 h before tetrad cell formation), meiosis (tetrad cells), auxospore formation and auxospore-resting spore formation and compared to vegetative growth stage. All the selected genes were significantly up-regulated at meiosis (>2-fold change), while at other stages of the sexual reproduction process the expression level of the six genes generally fluctuated within a smaller range (within +2 to −2) (Fig. 2).

### Flagellar gene distribution and phylogeny

To understand the evolution of genes coding for flagellum proteins in diatoms, we analysed the presence-absence of both specific (exclusively related to flagella, i.e., IFT and BBSome) and non-specific (also related to other functions) transcripts in diatom transcriptomes collected within the MMETSP project. The results definitely confirmed the complete absence of BBSome complexes in all diatoms and widened the list of IFT-B components, but interestingly also showed the presence of IFT-A components in many species (Table 1 and Additional file 8: Table S6 and Additional file 9: Table S7). In the pennate diatoms, we found few hits for IFT52 sequences for many species belonging to the classes Bacillariophyceae (including *Pseudo-nitzschia delicatissima* B596, *Nitzschia punctata* CCMP561, *Fragilariopsis kerguelensis* L26-C5 and *Amphiprora paludosa* CCMP125) and Fragilariophyceae (including *Thalassionema nitzschioides* L26-B, *Asterionellopsis glacialis* CCMP1581 and *Thalassiothrix antarctica* L6-D1) (Additional file 8: Table S6 and Additional file 9: Table S7). However, a reciprocal blastp indicated that these hits only retain an N-terminal domain containing ankyrin repeats and do not appear to be real IFT52 homologs. The finding of both IFT-A (IFT122 and IFT140) and IFT-B (IFT52, IFT80, IFT88 and IFT172) transcripts in the pennate *Pseudo*-*nitzschia fraudulenta* WWA7 (Additional file 9: Table S7) was instead unexpected. However, blast search revealed a high similarity of those transcripts to dinoflagellate sequences. Other transcripts related but not restricted to flagellar development or functions that were identified in *L. danicus* B650 were also retrieved in many centric diatom species except for dynein heavy chain axonemal, which was found only in *Ditylum brightwellii* Pop2 and *Proboscia alata* PI-D3. Many pennate diatoms were also found to have sequences similar to those non-specific genes (Additional file 9: Table S7).

**Table 1:** Comparative distribution of transcripts related to flagellar development belonging to IFT- A and IFT-B subcomplexes found in the centric diatom species (refer to supplementary Additional file 8: Tables S6 and Additional file 9: Table S7)

	IFT-A		IFT-B					
	IFT122	IFT140	IFT172	IFT52	IFT80	IFT88	TTC-26	TTC30a
*Attheya septentrionalis*				+				
*Aulacoseira subarctica*	+			+	+	+	+	+
*Chaetoceros curvisetus*				+		+	+	
*Corethron hystrix*				+				
*Corethron pennatum*				+		+		
*Coscinodiscus wailesii*			+	+	+	+	+	+
*Cyclotella meneghiniana*				+		+	+	+
*Dactyliosolen fragilissimus*				+	+			
*Ditylum brightwellii*	+		+	+	+	+	+	+
*Extubocellulus spinifer*			+	+		+		+
*Leptocylindrus aporus*				+	+		+	
*Leptocylindrus danicus*	+	+	+	+	+	+	+	+
*Leptocylindrus hargravesii*	+	+	+	+	+	+	+	
*Minutocellus polymorphus*				+	+	+		+
*Odontella aurita*	+		+	+	+	+	+	+
*Proboscia alata*	+	+	+	+	+	+	+	+
*Proboscia inermis*					+	+	+	
*Rhizosolenia setigera*	+	+	+	+		+		
*Skeletonema marinoi*				+		+		+
*Skeletonema menzelii*				+				+
*Stephanopyxis turris*	+		+	+	+	+	+	+
*Thalassiosira pseudonana* ^*a*^				+		+		+
*Thalassiosira rotula*				+		+		+
*Thalassiosira weissflogii*	+	+	+	+	+	+	+	+

^a^Based on genomic data [16]

**Fig. 3:**
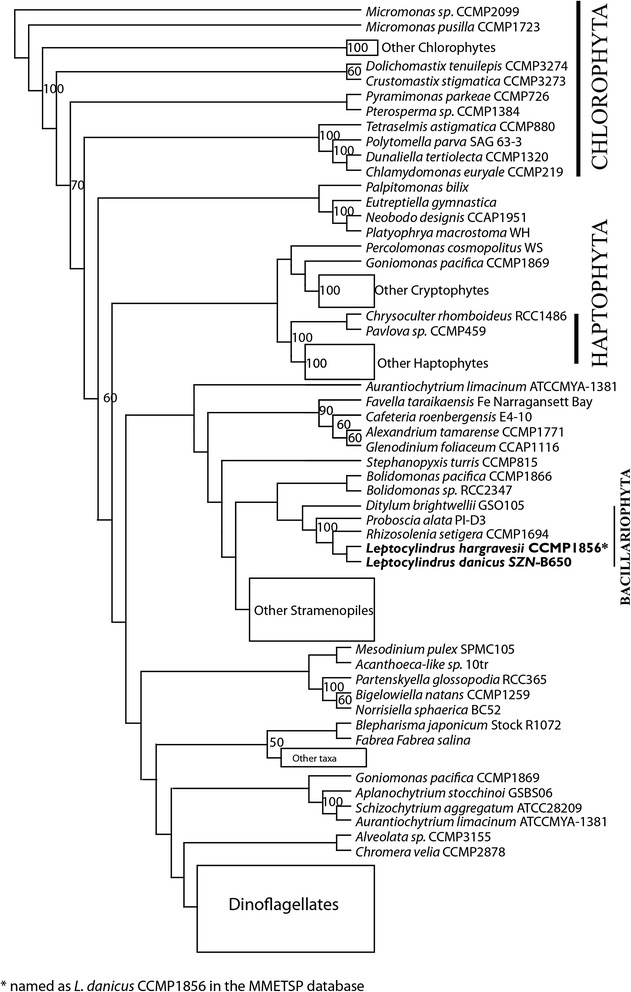
Maximum likelihood tree inferred from IFT172 peptide sequences illustrating the relationship among *Leptocylindrus* species and other protist groups. Bootstrap values have been generated with 1000 replicates

To understand the possible evolutionary origin and relationships in the genes encoding for the flagella, we investigated the relationships among the algal species utilising sequences retrieved from their transcriptomes for the IFT172 and IFT88 protein sequences. Maximum likelihood (ML) trees constructed using RAxML showed that the amino acid sequences of the IFT172 protein generally formed clades or grades reproducing known phylogenetic relationships in algal groups (Fig. 3, Additional file 10: Figure S6). Similar results were also obtained for the IFT88 protein, but with fewer taxa (Additional file 11: Figure S7).

## Discussion

### Flagellar gene in *L. danicus* during sexual reproduction

Although diatom transcriptome data are now available for many centric species, including those known to have sexual reproduction, the information on genes activated during sexual reproduction has so far been restricted to a few species for which specific experiments have been performed. In this respect, *L. danicus* is a special case of a species spontaneously undergoing sexual reproduction even in normal cultivation procedures, while other species may need specific induction procedures. A few flagellar genes had already been found in the genome of another centric diatom, *Thalassiosira pseudonana* [16], yet this first transcriptome study clearly shows the over representation of several flagellar genes in *L. danicus* compared to the congeneric species *L. aporus* not known to undergo sexual reproduction. Although the molecular functions of some of these genes and other associated genetic networks in the flagella development and sexual reproduction have not been fully elucidated, the present analysis suggests they are all involved in the flagellum development in male gametes.

In general, sex inducing factors in diatoms include decrease in cell size, environmental cues (for example temperature) and possible endogenous signaling molecules (sex pheromones) [6]. In *L. danicus,* as already reported [26], depleted nutrients (low N and Si) and lower growth temperature (16 °C) were found to be important factors that can stimulate sexualization. Nonetheless, like in other diatoms, getting laboratory cultures to undergo sexual reproduction may produce variable results, in terms of time taken for the process to occur and the magnitude at which it occurred, which probably depends on differences in the physiological status of the cells. In addition hallmarks of sexual reproduction in cultures grown under normal conditions, including the one used to obtain RNA for sequencing within the MMETSP project, indicate that sexual reproduction can occur in culture even uninduced.

The six genes chosen to explore expression in relation to sexual reproduction gene have been associated with specific processes related to flagellum development and functions. Cytoplasmic dynein 2 heavy chain, dynein heavy chain axonemal and intraflagellar transport protein 172 are three proteins involved in the bidirectional transport along axonemal microtubules, a process that is essential for the formation (ciliogenesis) and maintenance of most eukaryotic cilia and flagella [27, 28]. Tubulin tyrosine ligase-like 1 protein and tetratricopeptide repeat protein 30a are involved in the post-translational polyglutamylation of tubulin in axonemal microtubules [29, 30]. Finally, ‘B9 domain containing protein 1’ (which has a cilia-specific role) prevents diffusion of transmembrane proteins between the flagella and plasma membranes [31]. These genes were all found to be expressed in the early phases of the process corresponding to meiosis, and this is consistent with the notion that flagella develop in male gametes (sperms) of centric diatoms and specifically in *Leptocylindrus* [20, 21]. Additionally, our results indicate that there is only a narrow time window during sexual reproduction where flagellar genes are expressed.

### Flagellar genes distribution in diatoms and other protists

Since the transcriptomes of diatoms were not purposely obtained from cultures undergoing sexual reproduction, the absence of flagellum related-transcripts does not necessarily imply their absence in the genome of the species sequenced within the MMETSP. On the other hand, transcripts related to flagellar development in the transcriptome of several species for which sexual reproduction has not been described as yet (e.g., *Proboscia alata* and *Rhizosolenia setigera*) suggest that this process is widespread in diatoms. Their presence in so many cases might be due to background levels of transcription or, alternatively, to sexual reproduction occurring in the cultures undetected.

It is hypothesised that diatoms have lost both BBSome and IFT-A components during evolution, retaining only IFT-B components. In the current study, BBSome complexes were not found in any diatoms, confirming the hypothesis of their complete loss in this group. However, in contrast with the current hypothesis [16], the IFT-A subcomplex has been retained by centric diatoms, or at least by a number of them. In fact, *L. danicus*, *L. hargravesii*, *Proboscia alata, Rhizosolenia setigera,* and *Thalassiosira weissflogii* showed both IFT122 and IFT140, while *Aulacoseira subarctica, Odontella aurita, Stephanopyxis turris* and *Ditylum brightwellii* had IFT122 (Table 1 and Additional file 8: Tables S6 and Additional file 9: Table S7). Recently, IFT-A (IFT-140) was also reported in the brown algae *Colpomenia bullosa* and *Ectocarpus siliculosus*, which form flagellated gametes [32], corroborating the idea that IFT-A subcomplex is more widespread than thought so far. Within the IFT-B complex, many of the centric species were found to have IFT52, IFT80, IFT88 and IFT172 including *L. hargravesii* for which sexual reproduction has been reported [17].

While the centric diatoms contain both IFT-A and IFT-B subcomplexes, pennate diatoms do not seem to retain any of these proteins, which is consistent with the absence of flagellated gametes during sexual reproduction in these organisms. Indeed, the unexpected finding of IFT-A and IFT-B transcripts in the pennate diatom *Pseudo*-*nitzschia fraudulenta* WWA7 was eventually attributed to a contamination by a dinoflagellate in the culture submitted to MMETSP for transcriptome sequencing. Other transcripts related to flagellar functions were found in many pennate diatoms. However, because those proteins have additional roles, they do not represent a univocal indication of functional flagellum machinery.

Interestingly, the phylogenetic relationship for flagellar genes in protists displayed similar pattern as the ribosomal genes. For example, the diatoms species were found within the Stramenopiles and were sister to *Bolidomonas* spp., which matches what is generally seen in the phylogenetic trees based on ribosomal genes [17]. Thus it appears that the flagellar gene sequences are conserved within the groups and their evolution followed the general phylogenetic pattern expected from the tree of life.

## Conclusions

This study provided evidence at the molecular level for the functional differences among two closely related species *L. danicus* and *L. aporus,* otherwise showing remarkable morphological conservation and considered as a single species until recently [17, 33]. Among the GO terms that were differentially represented in the two species, those related to the flagellum, necessarily linked to the male gamete formation, represented a further evidence of the occurrence of sexual reproduction in *L. danicus* but not in *L. aporus*, while the gene expression levels variations for the first time demonstrated the activation of those genes over the sexual cycle.

The genes identified in this study based on the transcriptome of *L. danicus* remarkably widen the recognised flagellar gene set in this group of microbes. Considering the occurrence of flagellated cells only during gamete formation, those genes represent a clear hallmark of sexual reproduction, which will facilitate further investigations on this process in centric diatoms. In addition, the information provided about those genes can be used to detect signals of sexual reproduction of centric diatoms in the natural environment taking advantage of metatranscriptomic datasets that are being gathered in several investigations. This will shed light into the occurrence of a process otherwise hardly detected in nature, as well as into the timing of its occurrence with respect to the peak of the bloom and its actual frequency under different environmental conditions.

## Methods

### Cell culture

Fresh cultures of *L. danicus* strain SZN-B650 and *L. aporus* strain SZN-B651 (the strain was submitted as *L. danicus* strain SZN-B651 as only later it has become clear that it was a separate species, [17, 33]) were inoculated at a starting cell density of 200 cells ml^−1^ in 250 ml polystyrene culture flasks with 100 ml of Keller medium. Cultures were maintained at 20 °C with 100 μE m^−2^ s^−1^ light and a diurnal cycle of 12:12 h light: dark periods. The cultures were grown till they attained a cell density of 10^4^ cells ml^−1^ and later diluted to 1 L to give a cell density of 10^3^ cells ml^−1^. Then the cultures were further incubated under the same conditions for 3 more days. In this way the cells were always retained in the log phase while reducing the time of growth in larger volume. Once they attained the density of 10^4^ cells ml^−1^, the cultures were harvested by filtering on 1.2 μm pore-size nitrocellulose filters (Millipore, Italy). The circular filter was cut into four parts and each quarter was dipped in 1 ml of TRIzol®-reagent (Life Technologies Europe, Italy). The samples were either immediately processed or frozen in liquid nitrogen and stored at −80 °C for later RNA extraction.

### RNA extraction and sequencing

Frozen samples were thawed on ice and homogenised by vortexing for 2–3 min. The samples were then incubated on thermo shaker at maximum speed for 10 min at 60 °C, to help cell disruption and release of RNA. Subsequently, the samples were cooled to room temperature and 300 μl of chloroform was added under fume hood and was vigorously mixed by inverting the tubes. The tubes were incubated at room temperature for 15 min for complete precipitation. Thereafter, the samples were centrifuged at 10,000 *g* for 15 min at 4 °C. The aqueous supernatant (≈750 μL) above the white phase was carefully transferred to new RNase free pre-chilled 1.5 ml Eppendorf tube. Equal volume (750 μL) of isopropanol was added to the collected supernatant. The RNA in supernatant-isopropanol mix was allowed to precipitate under room temperature for 10 min. The mix was then centrifuged at 10,000 *g* for 10 min at 4 °C to pellet RNA. The obtained RNA pellet was washed once with 750 μL of 75% ethanol (in DEPC treated water), by centrifuging at 10,000 *g* for 10 min at 4 °C. Finally, the RNA pellet was resuspended in 50 μL of RNase free water and was stored in deep freezer.

Total RNA extracted using the above method was purified with an RNeasy kit (Qiagen), following the manufacturer’s protocol. Measures of RNA quality (purity and integrity) and abundance were determined for each sample using a NanoDrop Spectrophotometer (Thermo Fisher Scientific Inc., UK) to determine the A260/A280 ratio and RNA concentration, and the Agilent Bioanalyzer 2100 RNA Nano LabChip (Agilent, Palo Alto, CA) to generate an electropherogram and an RNA Integrity Number (RIN). A good quality RNA sample was used for total transcriptome sequencing.

Total RNA was submitted to the National Center for Genome Resources (NCGR, Santa Fe, NM) for sequencing under the project, Marine Microbial Eukaryote Transcriptome Project (MMETSP; Gordon and Betty Moore Foundation). RNA libraries were prepared from total RNA isolated using the standard Tru-Seq™ RNA protocol - poly-A+ selection (Illumina, USA) with an insert size of ~200 bp. Sequencing was done from both ends (paired-end reads 2 × 50 nt) on the Illumina Hi-Seq 2000 to obtain approximately 2.5 Gbp of data (*L. danicus*, MMETSP0321 and *L*. *aporus*, MMETSP0322). Reads were filtered for quality (Q15) and read length (0.5). The two transcriptomes can be downloaded from the iMicrobe Data Distribution Center [34], in which all MMETSP data have been deposited.

### Assembly and annotation

Sequences were assembled using AbySS [35] to generate 20 assemblies across a k-mer sweep from 26 to 50 nt. The obtained assemblies were then subjected to gap closing with GapCloser v 1.10 (Short Oligonucleotide Analysis Package, SOAPdenovo) [36]. Gap closed assemblies from the k-mer sweep were reconciled into one assembly using miraEST [37]. The assembled transcripts were annotated using blastx search in Blast2GO [38] against the NCBI non-redundant protein database with 10^−6^ e-value cut-off. Sequences were mapped and functionally annotated on Blast2GO with the default settings. GO functional classification for transcripts was performed using WEGO (Web Gene Ontology Annotation Plot) [39]. KEGG metabolic pathway annotation and COG classification of transcripts were determined through blastx search against KEGG database and COG database, respectively [40, 41]. The difference in GO term abundance between the two species was analysed using Fisher’s exact test with the GOSSIP package [42] implemented in Blast2GO, with support from *p*-value and subsequently the false detection rate (FDR) at 0.05.

### Inducing sexual reproduction

Meiosis was induced in cultures by reduction of the nutrient levels in the medium, designated as “T” medium (containing ammonia at 15 μM; phosphate at 7 μM; silicate at 50 μM; trace metals, vitamins, and Trizma as in f/2) [43], with at a slightly lower temperature of 16 °C, light of 80 μmol photons m^−2^ s^−1^ intensity and a 12:12 L:D cycle [26]. The strains submitted to these reduced nutrient conditions were observed regularly in the LM. On cultures where spore formation was observed, microscope pictures were taken and samples were collected at different stages for RNA extraction and real-time PCR analysis.

### Real-time PCR assays

Six annotated transcripts that may relate to functionally-conserved genes for flagella development in centric diatoms were selected and analysed using real-time PCR (specific primers are listed in Additional file 12: Table S8). For real-time PCR analysis, sexual reproduction in *Leptocylindrus danicus* culture was inoculated into a 200 ml polystyrene flask with 150 ml T media [44] to a cell density of 2000 cells ml^−1^ and incubated at 16 °C with 100 μE m^−2^ s^−1^ light and a photoperiod of 12:12 light:dark. Samples were collected at various stages of sexual reproduction and vegetative growth. Cultures were filtered and RNA was extracted following the same protocols described above. cDNA was synthesized from the extracted total RNA with SuperScript II RT (Invitrogen, USA), and quantitative real-time PCR was triply performed using the Applied Biosystems with a volume of 10 μL containing 1 μL of 1:5 cDNA diluted with RNase free water, 5 μL of 26SYBRGreen Master Mix, and 250 nM of each primer. The cycling parameters included 95 °C for 2 min, followed by 40 cycles of 95 °C for 1 s, 62 °C for 1 min, and 70 °C for 1 s. During each cycle fluorescence measurements were taken at 70 °C for 1 s. All the qPCR standardisations were performed using the cDNA prepared using cultures in vegetative growth. Expression levels of each gene for sexual reproduction were normalized to histone H4, transcription initiation factor subunit 12 and translation elongation factor 1 alpha. Statistical analysis was performed with the relative quantification method described by Muller et al. [45].

### Homology based searches

tblastn searches were conducted using *L. danicus* protein sequences as queries against the nucleotide collection of MMETSP currently available. Only hits with an e-value smaller or equal to 1e-10 were considered. Blast results for diatoms only are shown in Additional file 7: Table S5. Peptide sequences for selected protein hits were retrieved from iMicrobe [34] and blasted in public databases (blastp in nr) to confirm protein identity.

### Phylogenetic analysis

To study the evolutionary pattern of the flagellum coding genes in diatoms and other algae for which the sequence information is available, we applied maximum likelihood relationship approach. Sequences for the IFT172 and IFT88 genes involved in the flagellum development identified in *L. danicus* were downloaded from NCBI nucleotide database through homology searches using blastn. Sequences were also obtained by local BLAST searches from the transcript dataset that constituted many publically available transcriptomes of species sequenced within the Marine microbial eukaryotic transcriptome sequencing project (e-value smaller or equal to 1e-10). Obtained sequences were gathered into datasets and sequence alignment was generated using MUSCLE version 3.8.31 [46]. Fewer taxa were used for IFT88 than IFT172 analysis. The IFT172 dataset was comprised of 137 sequences which were aligned to give an alignment length of 2732 bp. The IFT88 dataset included 99 sequences that were aligned with an alignment length of 1324 bp. Any misalignments were corrected manually and phylogenies for each of the dataset was constructed following a maximum-likelihood approach [47] using the WAG amino acid substitution model [48] with a discrete gamma distribution [49] and nonparametric bootstrap of 1000 replicates.

## Additional files

Additional file 1: Sequence assembly and annotation, and **Table S1** **-** **Table S4**. **Table S1**. Summary of sequence assembly and sequence statistics for the transcriptomes of *L. danicus* and *L. aporus*. B1000 and B2000 indicate the percentage of bases involved in contigs of at least 1000 bp and 2000 bp, respectively. **Table S2**. GO classification of annotated contigs derived from illumina sequencing of the species *L. danicus* and *L. aporus*. 7330 and 4914 annotated sequences of *L. danicus* and *L. aporus* respectively, were assigned to 6261and 5390 GO categories, and the terms were summarized into three main categories and 51 subcategories. (MF: 1446, 1267; BP: 4199, 3568; CC: 616, 555). **Table S3**. KOG function classification of the *L. danicus* and *L. aporus* contigs. 13,723 and 8949 transcripts showed homology to KOG database at NCBI, classified among the 26 categories. **Table S4**. Kyoto Encyclopedia of Genes and Genomes (KEGG) classification of *Leptocylindrus danicus* and *L. aporus* transcripts. At a cutoff e-value of <1e¬5, 5917 transcripts of *L. danicus* and 4645 transcripts of *L. aporus* were assigned to 388 and 298 pathways respectively. (DOCX 25 kb)

Additional file 2: Figure S1. Data distribution of *L. danicus* and *L. aporus* transcriptome after BLAST hits against NR protein database. The figure shows the absolute number of sequences with or without blast hits and how many were annotated and mapped. (TIFF 589 kb)

Additional file 3: Figure S2. Species distribution of *L. danicus* and *L. aporus* transcriptome after BLAST hits against NCBI NR protein database. A total of 18,315 and 12,312 contigs had protein hits covering 60.35% and 69.26% of the transcriptome for *L. danicus* and *L. aporus*, respectively. (TIFF 1300 kb)

Additional file 4: Figure S3. WEGO functional classification of assembled *L. danicus* and *L. aporus* transcripts. Results are summarized for three main GO categories: biological process, cellular component and molecular function. (TIFF 1284 kb)

Additional file 5: Figure S4. Pie charts for GO functional classification of assembled *L. danicus* and *L. aporus* transcripts. Results are summarized as pie charts for three main GO categories: biological process, cellular component and molecular function. 7330 transcripts of *L. danicus* were assigned to 6261 GO terms and 4914 transcripts of *L. aporus* were assigned to 5390 GO categories. (TIFF 1078 kb)

Additional file 6: Figure S5. KOG functional classification of assembled *L. danicus* and *L. aporus* transcripts based on homology searches at e-value cut-off of 0.001. The obtained results were classified into 26 functional categories from A to Z. (TIFF 1263 kb)

Additional file 7: Table S5. List of GO terms that were over or under represented in the transcriptome of the species *L. danicus* and *L. aporus.* Comparison was made on Fischer excat test in Blast2GO and only GO terms that are significantly different are listed (*p*-value <0.05). (XLSX 179 kb)

Additional file 8: Table S6. Comparative presence or absence of all flagellar genes in diatom species showing at least one IFT-A or IFT-B subcomplex transcripts. For an exhaustive list, see Table S7. (XLSX 14 kb)

Additional file 9: Table S7. Presence of flagellar genes found in the transcriptome of *L. danicus* blast searched in other diatom species sequenced in the MMTESP project. The taxonomy is reported as in the original database, despite some obvious mistakes (e.g. one strain of *Minutocellus*, *Chaetoceros brevis* and *C. dichaeta* under Bacillariophyceae). (XLSX 216 kb)

Additional file 10: Figure S6. Uncollapsed Maximum Likelihood tree inferred from IFT172 peptide sequences illustrating the relationship among *Leptocylindrus* species and other protist groups. Bootstrap values have been generated with 1000 replicates. (TIFF 2938 kb)

Additional file 11: Figure S7. Maximum likelihood tree inferred from IFT88 peptide sequences illustrating the relationship among *Leptocylindrus* species and other protist groups. Sequences for the gene identified in the *L. danicus* were blastn searched from MMETSP identified and also downloaded from NCBI nucleotide database Bootstrap values have been generated with 1000 replicates. (TIFF 940 kb)

Additional file 12: Table S8. Sequences of the forward and reverse primers for six selected flagella genes and three reference genes analysed using qPCR. Gene, gene description and primer sequences are listed. (XLSX 9 kb)

## Abbreviations

BBSome: Bardet–Biedl syndrome
FDR: False detection rate
GO: Gene ontology
IFT: IntraFlagellar transport
KEGG: Kyoto Encyclopedia of Genes and Genomes
KOG: Eukaryotic Orthologous Groups of proteins
MMETSP: Marine Microbial Eukaryote Transcriptome Sequencing Project
NCGR: National Center for Genome Resources
WEGO: Web Gene Ontology Annotation Plot
