# Supplementary material for: Diatom flagellar genes and their expression during sexual reproduction in Leptocylindrus danicus
Source: BMC Genomics. 2017 Oct 23;18:813. doi: 10.1186/s12864-017-4210-8 (PMC5654045; doi:10.1186/s12864-017-4210-8)
Supplement: Supplementary file 1 — Sequence assembly and annotation, and Table S1 - Table S4. Table S1. Summary of sequence assembly and sequence statistics for the transcriptomes of L. danicus and L. aporus. B1000 and B2000 indicate the percentage of bases involved in contigs of at least 1000 bp and 2000 bp, respectively. Table S2. GO classification of annotated contigs derived from illumina sequencing of the species L. danicus and L. aporus. 7330 and 4914 annotated sequences of L. danicus and L. aporus respectively, were assigned to 6261and 5390 GO categories, and the terms were summarized into three main categories and 51 subcategories. (MF: 1446, 1267; BP: 4199, 3568; CC: 616, 555). Table S3. KOG function classification of the L. danicus and L. aporus contigs. 13,723 and 8949 transcripts showed homology to KOG database at NCBI, classified among the 26 categories. Table S4. Kyoto Encyclopedia of Genes and Genomes (KEGG) classification of Leptocylindrus danicus and L. aporus transcripts. At a cutoff e-value of <1e¬5, 5917 transcripts of L. danicus and 4645 transcripts of L. aporus were assigned to 388 and 298 pathways respectively. (DOCX 25 kb) [file 12864_2017_4210_MOESM1_ESM.docx]

Table S1. Summary of sequence assembly and sequence statistics for the transcriptomes of *L. danicus* and *L. aporus*. B1000 and B2000 indicate the percentage of bases involved in contigs of at least 1000 bp and 2000 bp, respectively.

|  | *L. danicus* | *L. aporus* |
| --- | --- | --- |
| Number of sequences | 30,346 | 17,776 |
| Total number of characters | 37,800,254 | 26,282,850 |
| Range sequence length (bp) | 150–29,076 | 150–16,580 |
| N50 | 1969 | 1982 |
| B1000 (%) | 80.1 | 85.8 |
| B2000 (%) | 40.2 | 49.4 |

Table S2. GO classification of annotated contigs derived from illumina sequencing of the species *L. danicus* and *L. aporus*. 7330 and 4914 annotated sequences of *L. danicus* and *L. aporus* respectively, were assigned to 6261and 5390 GO categories, and the terms were summarized into three main categories and 51 subcategories. (MF: 1446, 1267; BP: 4199, 3568; CC: 616, 555)

| GO level 2 classification | Number of genes associated | | percentage of the associated gene number to the total annotated contigs | | Pearson Chi-Square test between the gene numbers |
| --- | --- | --- | --- | --- | --- |
| Cellular Component | ***L. danicus*** | ***L. aporus*** | **% *L. danicus*** | **% *L. aporus*** | **p-value** |
| GO:0005576 extracellular region | 42 | 36 | 0.1 | 0.2 | 0.092 |
| GO:0044421 extracellular region part | 10 | 4 | 0 | 0 | 0.516 |
| GO:0005623 cell | 3208 | 2183 | 10.6 | 12.3 | 0 |
| GO:0044464 cell part | 3208 | 2182 | 10.6 | 12.3 | 0 |
| GO:0031974 membrane-enclosed lumen | 286 | 226 | 0.9 | 1.3 | 0.001 |
| GO:0031975 envelope | 150 | 111 | 0.5 | 0.6 | 0.061 |
| GO:0032991 macromolecular complex | 790 | 554 | 2.6 | 3.1 | 0.001 |
| GO:0043226 organelle | 1970 | 1316 | 6.5 | 7.4 | 0 |
| GO:0044422 organelle part | 932 | 642 | 3.1 | 3.6 | 0.001 |
| GO:0044421 extracellular region part | 10 | 4 | 0 | 0 | 0.516 |
| GO:0044422 organelle part | 932 | 642 | 3.1 | 3.6 | 0.001 |
| GO:0044456 synapse part | 1 | 1 | 0 | 0 | Ml |
| GO:0044464 cell part | 3208 | 2182 | 10.6 | 12.3 | 0 |
| GO:0045202 synapse | 2 | 3 | 0 | 0 | Ml |
| GO:0044456 synapse part | 1 | 1 | 0 | 0 | Ml |
| GO:0055044 symplast | 42 | 32 | 0.1 | 0.2 | 0.261 |
| Total cellular component:16 |  |  |  |  |  |
|  |  |  |  |  |  |
| Biological Process |  |  |  |  |  |
| GO:0000003 reproduction | 168 | 124 | 0.6 | 0.7 | 0.05 |
| GO:0044085 cellular component biogenesis | 241 | 158 | 0.8 | 0.9 | 0.269 |
| GO:0032502 developmental process | 538 | 336 | 1.8 | 1.9 | 0.352 |
| GO:0016043 cellular component organization | 547 | 318 | 1.8 | 1.8 | 0.914 |
| GO:0016265 death | 87 | 54 | 0.3 | 0.3 | 0.738 |
| GO:0022414 reproductive process | 163 | 114 | 0.5 | 0.6 | 0.145 |
| GO:0002376 immune system process | 104 | 64 | 0.3 | 0.4 | 0.756 |
| GO:0050896 response to stimulus | 677 | 494 | 2.2 | 2.8 | 0 |
| GO:0032501 multicellular organismal process | 551 | 369 | 1.8 | 2.1 | 0.044 |
| GO:0010926 anatomical structure formation | 214 | 121 | 0.7 | 0.7 | 0.755 |
| GO:0051704 multi\-organism process | 94 | 78 | 0.3 | 0.4 | 0.022 |
| GO:0051234 establishment of localization | 741 | 504 | 2.4 | 2.8 | 0.009 |
| GO:0022610 biological adhesion | 15 | 9 | 0 | 0.1 | 0.955 |
| GO:0008152 metabolic process | 3720 | 2589 | 12.3 | 14.6 | 0 |
| GO:0016032 viral reproduction | 29 | 22 | 0.1 | 0.1 | 0.359 |
| GO:0048511 rhythmic process | 14 | 4 | 0 | 0 | 0.196 |
| GO:0043473 pigmentation | 773 | 513 | 2.5 | 2.9 | 0.026 |
| GO:0040011 locomotion | 49 | 25 | 0.2 | 0.1 | 0.574 |
| GO:0051179 localization | 791 | 534 | 2.6 | 3 | 0.01 |
| GO:0040007 growth | 76 | 61 | 0.3 | 0.3 | 0.065 |
| GO:0009987 cellular process | 3665 | 2549 | 12.1 | 14.3 | 0 |
| GO:0001906 cell killing | 1 | 0 | 0 | 0 | Ml |
| GO:0065007 biological regulation | 880 | 585 | 2.9 | 3.3 | 0.016 |
| Total Biological Process:23 |  |  |  |  |  |
|  |  |  |  |  |  |
| Molecular Function |  |  |  |  |  |
| GO:0009055 electron carrier activity | 58 | 38 | 0.2 | 0.2 | 0.591 |
| GO:0060089 molecular transducer activity | 43 | 38 | 0.1 | 0.2 | 0.063 |
| GO:0030528 transcription regulator activity | 96 | 49 | 0.3 | 0.3 | 0.432 |
| GO:0030234 enzyme regulator activity | 69 | 35 | 0.2 | 0.2 | 0.487 |
| GO:0003824 catalytic activity | 4294 | 2958 | 14.2 | 16.6 | 0 |
| GO:0005488 binding | 3247 | 2257 | 10.7 | 12.7 | 0 |
| GO:0016209 antioxidant activity | 20 | 10 | 0.1 | 0.1 | 0.682 |
| GO:0015457 auxiliary transport protein activity | 3 | 1 | 0 | 0 | Ml |
| GO:0016530 metallochaperone activity | 2 | 2 | 0 | 0 | Ml |
| GO:0045182 translation regulator activity | 78 | 56 | 0.3 | 0.3 | 0.244 |
| GO:0005198 structural molecule activity | 228 | 136 | 0.8 | 0.8 | 0.867 |
| GO:0005215 transporter activity | 329 | 193 | 1.1 | 1.1 | 0.987 |
| Total Molecular Function:12 | | | | | |
| Total GO terms in three ontologies: 51 | | | |  |  |

MI = MeaningLess, when the expected counts are less than 5.

Table S3. KOG function classification of the *L. danicus* and *L. aporus* contigs. 13,723 and 8,949 transcripts showed homology to KOG database at NCBI, classified among the 26 categories.

|  | *L. danicus* | *L. aporus* | %*L. danicus* | %*L. aporus* |
| --- | --- | --- | --- | --- |
| A: RNA processing and modification | 638 | 426 | 4.65 | 4.76 |
| B: Chromatin structure and dynamics | 311 | 170 | 2.27 | 1.90 |
| C: Energy production and conversion | 446 | 350 | 3.25 | 3.91 |
| D: Cell cycle control, cell division, chromosome partitioning | 399 | 236 | 2.91 | 2.64 |
| E: Amino acid transport and metabolism | 536 | 433 | 3.91 | 4.84 |
| F: Nucleotide transport and metabolism | 128 | 92 | 0.93 | 1.03 |
| G: Carbohydrate transport and metabolism | 422 | 343 | 3.08 | 3.83 |
| H: Coenzyme transport and metabolism | 181 | 128 | 1.32 | 1.43 |
| I: Lipid transport and metabolism | 446 | 325 | 3.25 | 3.63 |
| J: Translation, ribosomal structure and biogenesis | 730 | 494 | 5.32 | 5.52 |
| K: Transcription | 1164 | 691 | 8.48 | 7.72 |
| L: Replication, recombination and repair | 489 | 240 | 3.56 | 2.68 |
| M: Cell wall/membrane/envelope biogenesis | 147 | 160 | 1.07 | 1.79 |
| N: Cell motility | 5 | 2 | 0.04 | 0.02 |
| O: Posttranslational modification, protein turnover, chaperones | 1330 | 1032 | 9.69 | 11.53 |
| P: Inorganic ion transport and metabolism | 430 | 278 | 3.13 | 3.11 |
| Q: Secondary metabolites biosynthesis, transport and catabolism | 237 | 162 | 1.73 | 1.81 |
| R: General function prediction only | 1629 | 975 | 11.87 | 10.90 |
| S: Function unknown | 778 | 493 | 5.67 | 5.51 |
| T: Signal transduction mechanisms | 1527 | 914 | 11.13 | 10.21 |
| U: Intracellular trafficking, secretion, and vesicular transport | 754 | 502 | 5.49 | 5.61 |
| V: Defense mechanisms | 56 | 25 | 0.41 | 0.28 |
| W: Extracellular structures | 69 | 41 | 0.50 | 0.46 |
| X: multiple functions | 0 | 1 | 0.00 | 0.01 |
| Y: Nuclear structure | 86 | 51 | 0.63 | 0.57 |
| Z: Cytoskeleton | 785 | 385 | 5.72 | 4.30 |
|  | **13723** | **8949** |  |  |

Table S4: KEGG classification of *Leptocylindrus danicus* and *L. aporus* transcripts.

| KEGG categories | Number of genes | | Number of sequences | |
| --- | --- | --- | --- | --- |
|  | ***L. danicus*** | ***L. aporus*** | ***L. danicus*** | ***L. aporus*** |
| Metabolism | **1023** | **967** | **1785** | **1583** |
| Carbohydrate metabolism | 187 | 175 | 360 | 332 |
| Energy metabolism | 124 | 121 | 223 | 214 |
| Lipid metabolism | 108 | 106 | 173 | 169 |
| Nucleotide metabolism | 131 | 112 | 205 | 160 |
| Amino acid metabolism | 190 | 179 | 345 | 287 |
| Metabolism of other amino acids | 47 | 47 | 89 | 81 |
| Glycan biosynthesis and metabolism | 41 | 40 | 62 | 58 |
| Metabolism of cofactors and vitamins | 103 | 94 | 176 | 139 |
| Metabolism of terpenoids and polyketides | 34 | 35 | 46 | 49 |
| Biosynthesis of other secondary metabolites | 24 | 21 | 46 | 35 |
| Xenobiotics biodegradation and metabolism | 34 | 37 | 60 | 59 |
| Genetic Information Processing | **726** | **688** | **1157** | **992** |
| Transcription | 123 | 117 | 193 | 153 |
| Translation | 264 | 261 | 434 | 385 |
| Folding, sorting and degradation | 204 | 197 | 318 | 295 |
| Replication and repair | 135 | 113 | 212 | 159 |
| Environmental Information Processing | **186** | **163** | **389** | **294** |
| Membrane transport | 9 | 8 | 19 | 13 |
| Signal transduction | 174 | 154 | 363 | 280 |
| Signaling molecules and interaction | 3 | 1 | 7 | 1 |
| Cellular Processes | **347** | **260** | **677** | **409** |
| Transport and catabolism | 129 | 114 | 241 | 185 |
| Cell motility | 10 | 7 | 21 | 13 |
| Cell growth and death | 181 | 116 | 350 | 163 |
| Cell communication | 27 | 23 | 65 | 48 |
| Organismal Systems | **352** | **291** | **702** | **448** |
| Immune system | 64 | 52 | 100 | 67 |
| Endocrine system | 89 | 78 | 183 | 127 |
| Circulatory system | 11 | 9 | 23 | 15 |
| Digestive system | 34 | 25 | 73 | 39 |
| Excretory system | 29 | 24 | 50 | 39 |
| Nervous system | 79 | 65 | 170 | 102 |
| Sensory system | 10 | 8 | 31 | 11 |
| Development | 10 | 7 | 16 | 9 |
| Environmental adaptation | 26 | 23 | 56 | 39 |
| Human Diseases | **659** | **584** | **1207** | **919** |
| Cancers: Overview | 82 | 71 | 165 | 121 |
| Cancers: Specific types | 72 | 55 | 110 | 84 |
| Immune diseases | 24 | 24 | 37 | 48 |
| Neurodegenerative diseases | 139 | 128 | 222 | 168 |
| Substance dependence | 25 | 21 | 65 | 46 |
| Cardiovascular diseases | 10 | 10 | 24 | 18 |
| Endocrine and metabolic diseases | 34 | 32 | 52 | 39 |
| Infectious diseases: Bacterial | 83 | 75 | 170 | 120 |
| Infectious diseases: Viral | 171 | 154 | 322 | 251 |
| Infectious diseases: Parasitic | 19 | 14 | 40 | 24 |
| Total | **3293** | **2953** | **5917** | **4645** |
