# Supplementary figures and images for: Diatom flagellar genes and their expression during sexual reproduction in Leptocylindrus danicus
Source: BMC Genomics. 2017 Oct 23;18:813. doi: 10.1186/s12864-017-4210-8 (PMC5654045; doi:10.1186/s12864-017-4210-8)

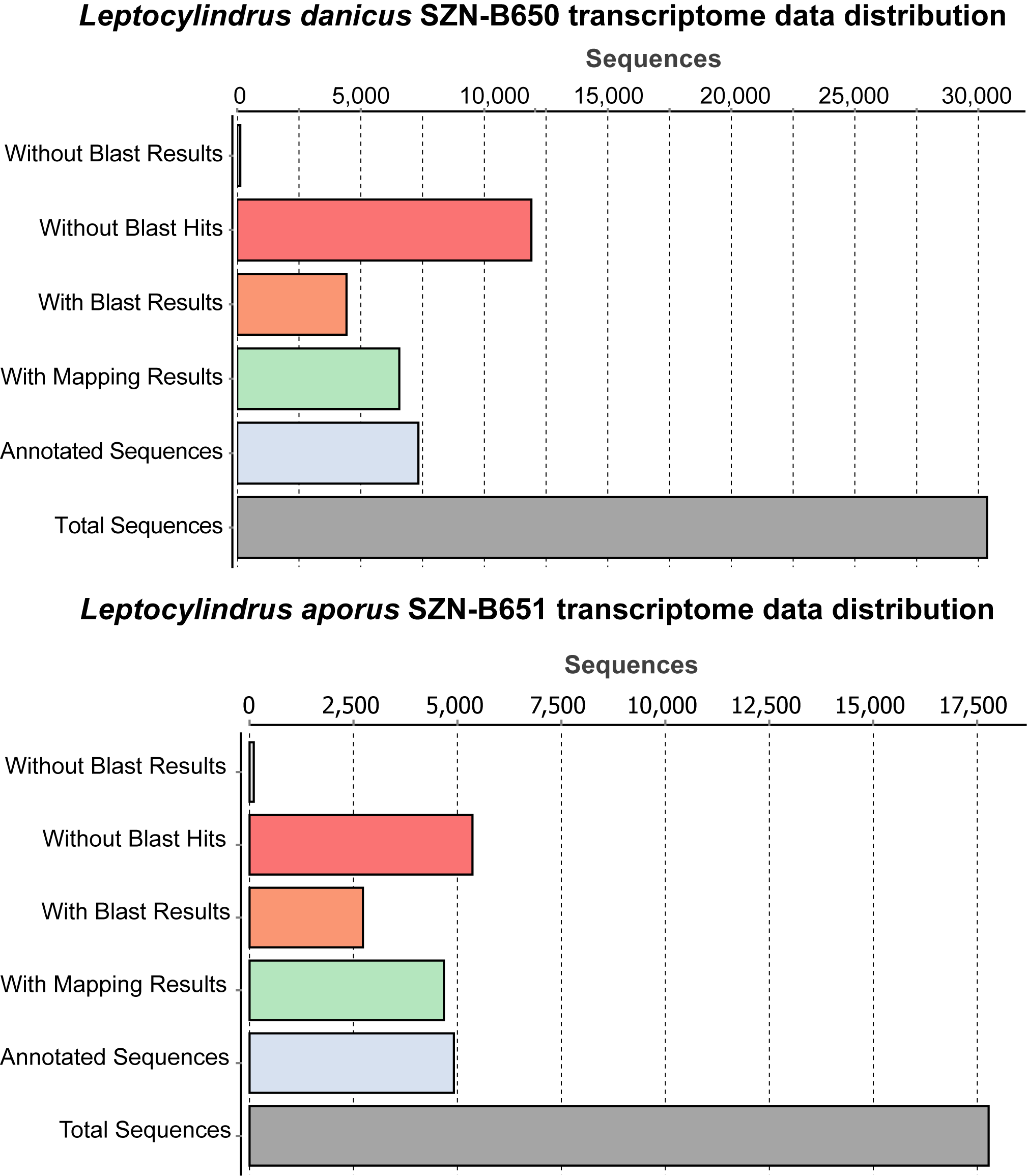

Supplement: Supplementary file 2 — Data distribution of L. danicus and L. aporus transcriptome after BLAST hits against NR protein database. The figure shows the absolute number of sequences with or without blast hits and how many were annotated and mapped. (TIFF 589 kb) [file 12864_2017_4210_MOESM2_ESM.tif]

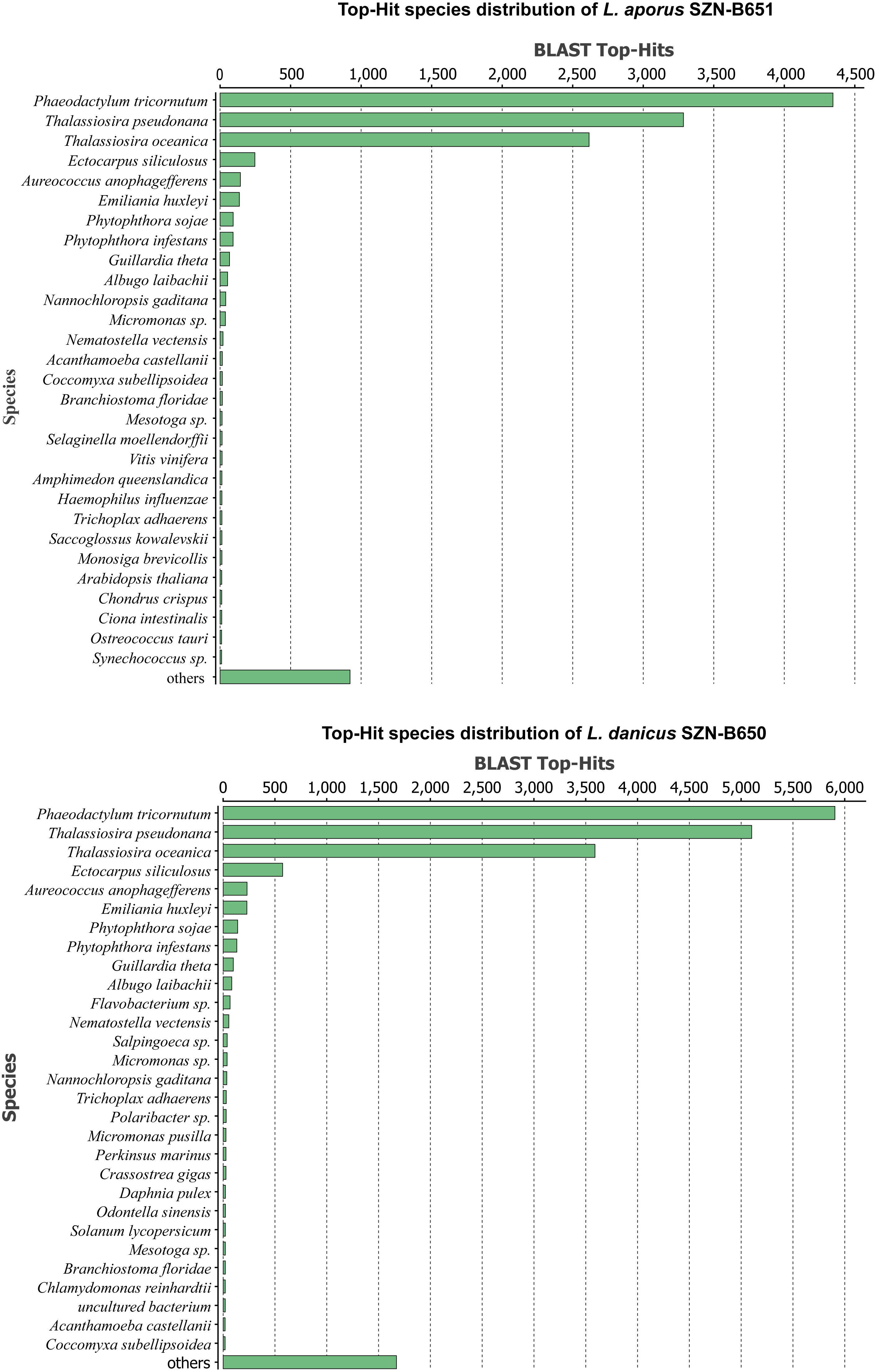

Supplement: Supplementary file 3 — Species distribution of L. danicus and L. aporus transcriptome after BLAST hits against NCBI NR protein database. A total of 18,315 and 12,312 contigs had protein hits covering 60.35% and 69.26% of the transcriptome for L. danicus and L. aporus, respectively. (TIFF 1300 kb) [file 12864_2017_4210_MOESM3_ESM.tif]

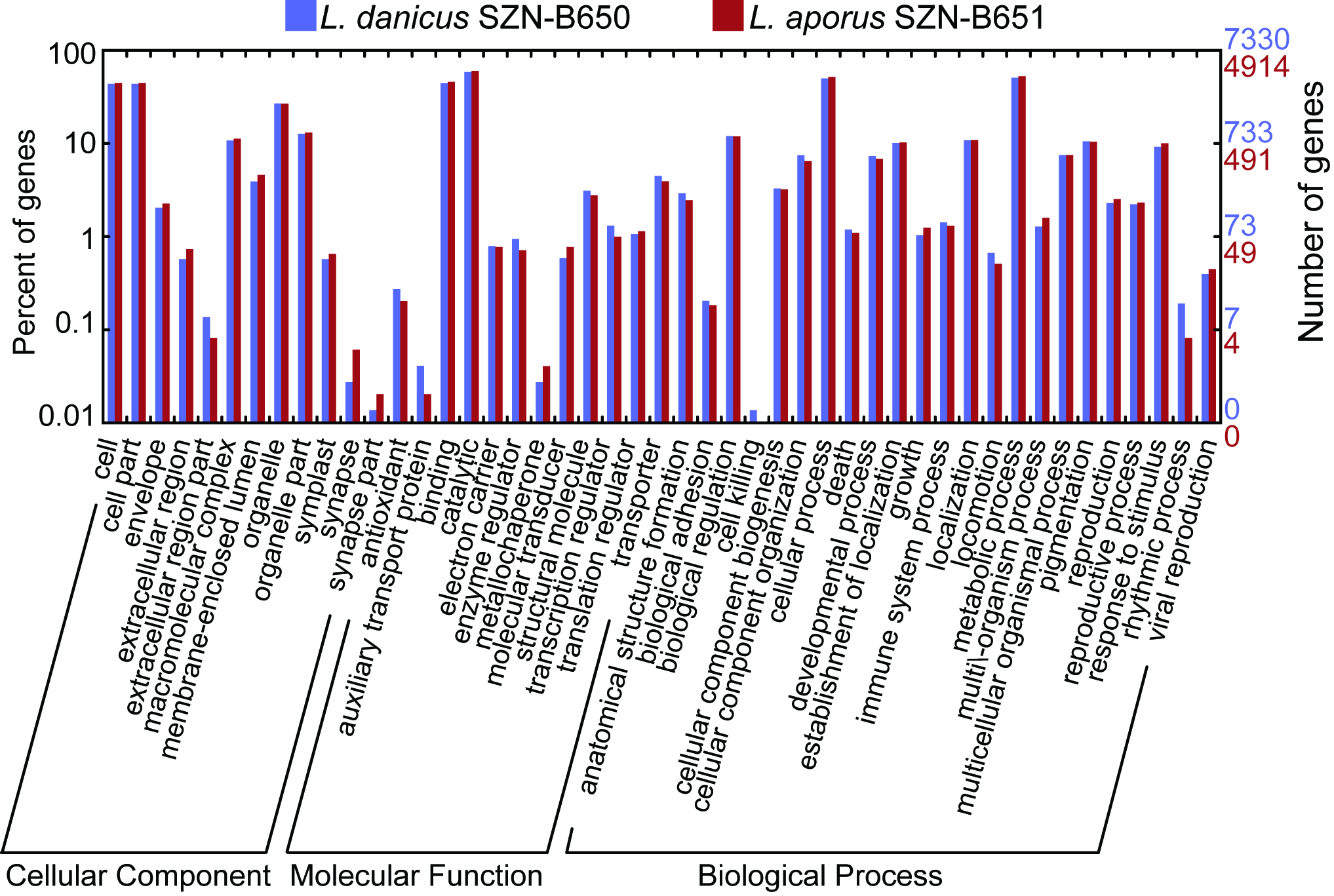

Supplement: Supplementary file 4 — WEGO functional classification of assembled L. danicus and L. aporus transcripts. Results are summarized for three main GO categories: biological process, cellular component and molecular function. (TIFF 1284 kb) [file 12864_2017_4210_MOESM4_ESM.tif]

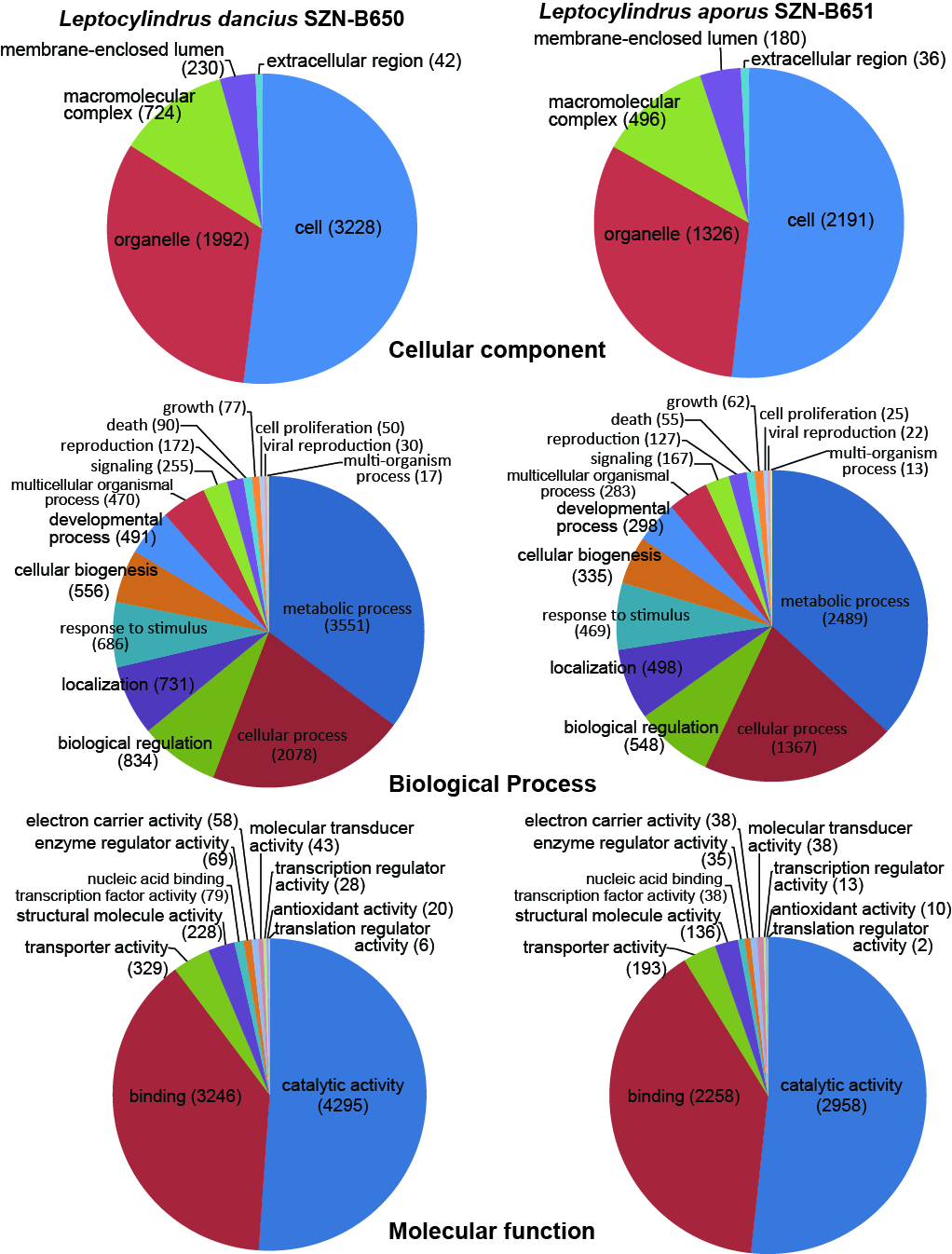

Supplement: Supplementary file 5 — Pie charts for GO functional classification of assembled L. danicus and L. aporus transcripts. Results are summarized as pie charts for three main GO categories: biological process, cellular component and molecular function. 7330 transcripts of L. danicus were assigned to 6261 GO terms and 4914 transcripts of L. aporus were assigned to 5390 GO categories. (TIFF 1078 kb) [file 12864_2017_4210_MOESM5_ESM.tif]

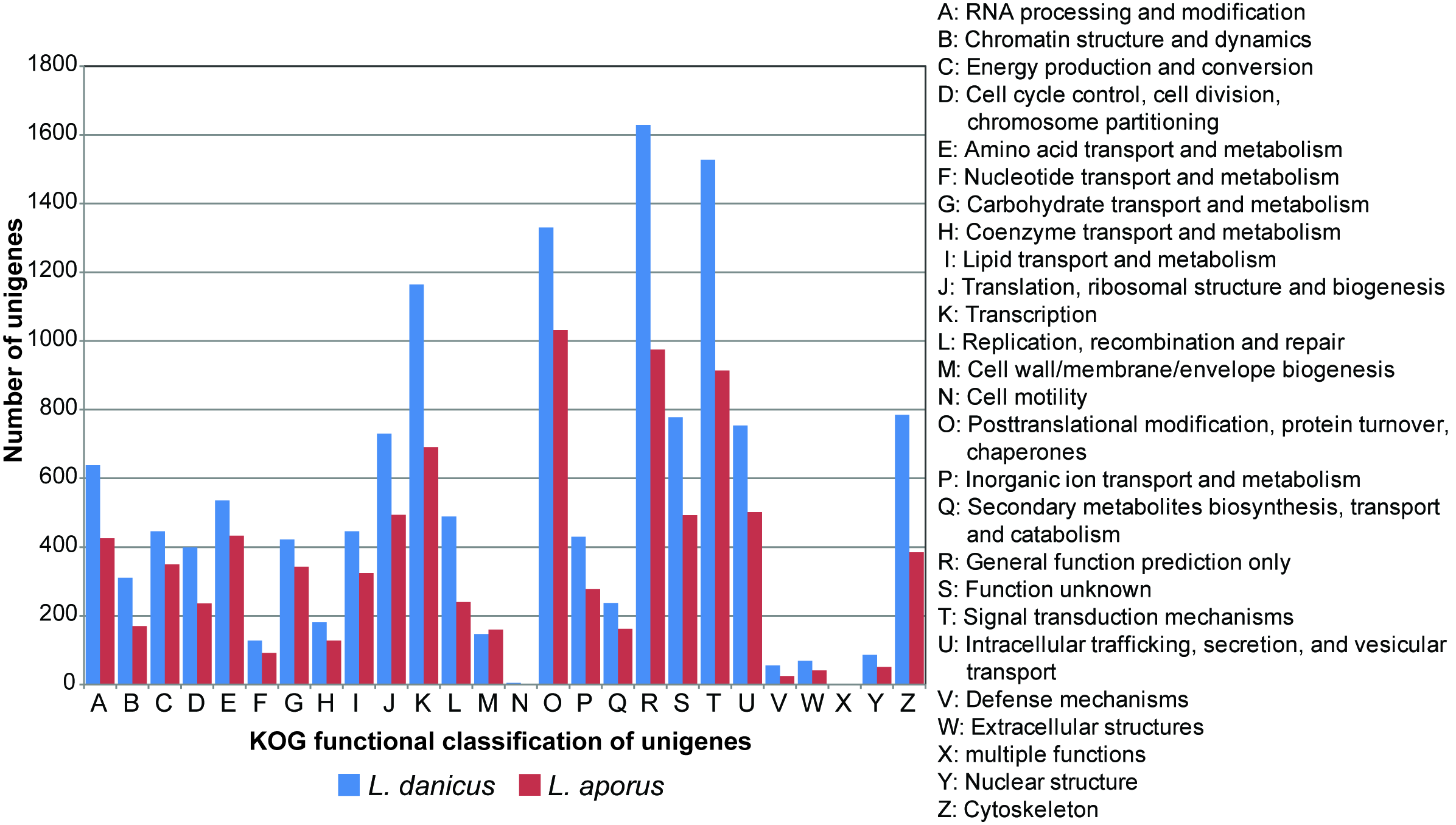

Supplement: Supplementary file 6 — KOG functional classification of assembled L. danicus and L. aporus transcripts based on homology searches at e-value cut-off of 0.001. The obtained results were classified into 26 functional categories from A to Z. (TIFF 1263 kb) [file 12864_2017_4210_MOESM6_ESM.tif]

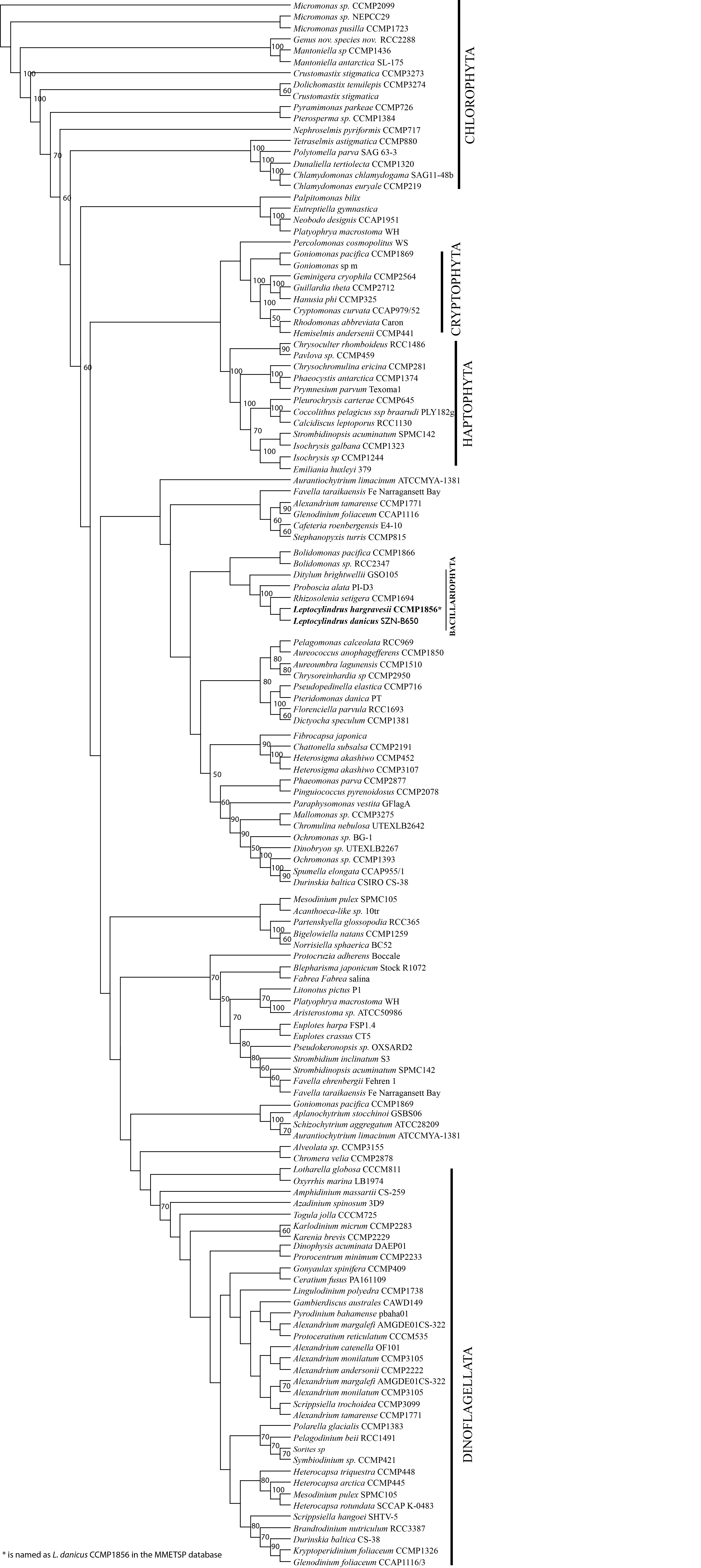

Supplement: Supplementary file 10 — Uncollapsed Maximum Likelihood tree inferred from IFT172 peptide sequences illustrating the relationship among Leptocylindrus species and other protist groups. Bootstrap values have been generated with 1000 replicates. (TIFF 2938 kb) [file 12864_2017_4210_MOESM10_ESM.tif]

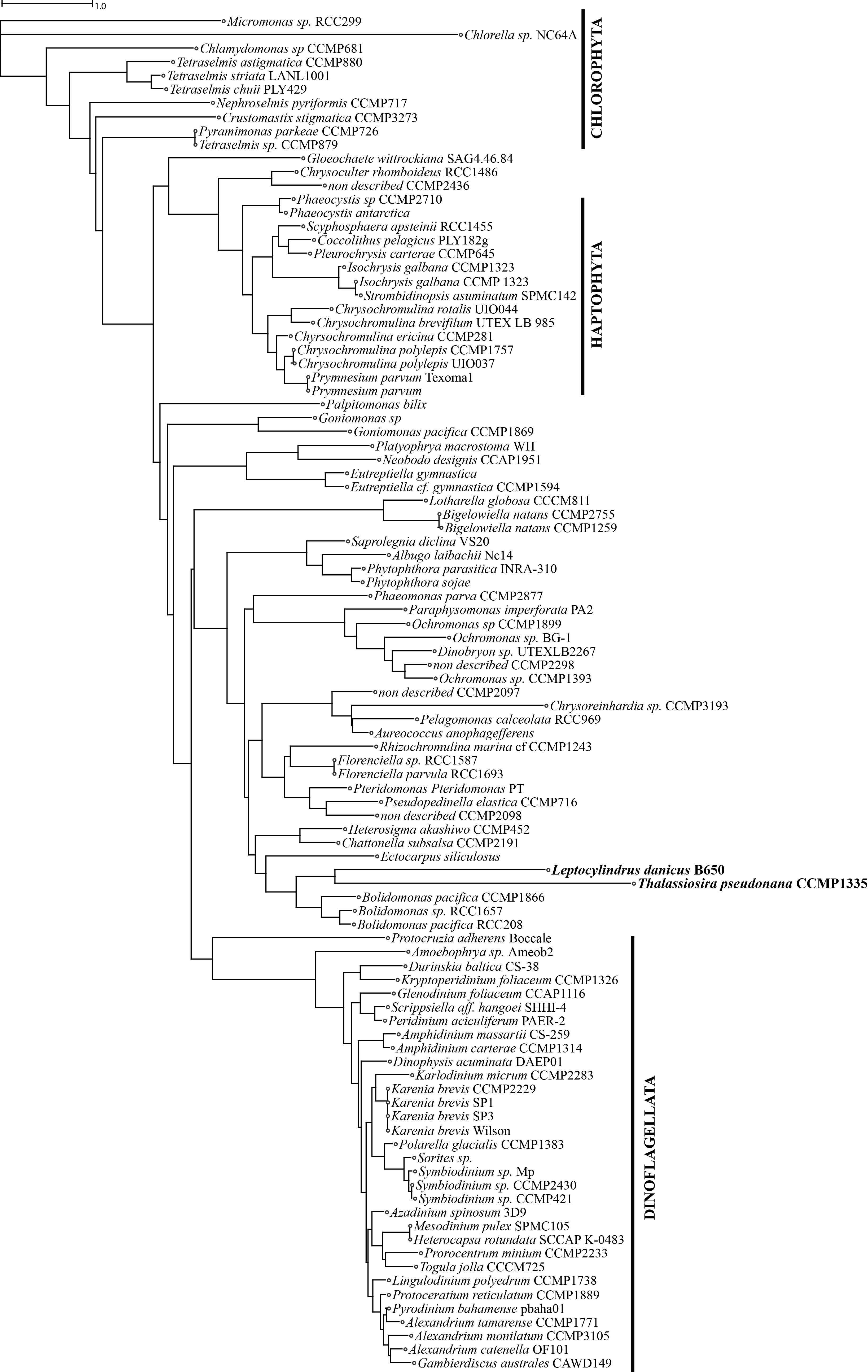

Supplement: Supplementary file 11 — Maximum likelihood tree inferred from IFT88 peptide sequences illustrating the relationship among Leptocylindrus species and other protist groups. Sequences for the gene identified in the L. danicus were blastn searched from MMETSP identified and also downloaded from NCBI nucleotide database Bootstrap values have been generated with 1000 replicates. (TIFF 940 kb) [file 12864_2017_4210_MOESM11_ESM.tif]
